# Accuracy of 3D digital modeling of dental arches

**DOI:** 10.1590/2177-6709.24.1.38.e1-7.onl

**Published:** 2019

**Authors:** Riccardo Favero, Andrea Volpato, Maurizio De Francesco, Adolfo Di Fiore, Riccardo Guazzo, Lorenzo Favero

**Affiliations:** 1 Private practice (Treviso, Italy).; 2 Università Degli Studi di Padova, Instituto di Clinica Odontoiatrica, Dipartimento di Neuroscienze (Padova, Italy).

**Keywords:** Digital impression, Intraoral scanner, Scanning technique

## Abstract

**Objective:**

The aim of the study was to verify and compare the accuracy of full-arch digital impressions obtained using two intraoral scanners and three scanning methodologies.

**Methods:**

A resin model created with dental 3-D printing was scanned by a reference scanner (Zfx Evolution - Zimmer Biomet, Palm Beach Gardens, FL) in order to obtain a 3D reference; the same resin model was then scanned with two different intraoral scanners (Zfx IntraScan and Carestream 3600 - CS 3600^®^, Carestream, Rochester, NY, USA) using: Technique A (from tooth #27 up to tooth #17); Technique B (from tooth #11 up to tooth #17 and then from tooth #21 up to tooth #27) and Technique C (from tooth #22 up to tooth #17, and then from tooth #12 up to tooth #27 - the MeshLab software v. 1.3.3 was then used to match the two scans). The scans obtained were superimposed over the reference scan by means of a software, and the volumetric discrepancies were calculated.

**Results:**

The mean results for the Zfx Intrascan scanner were: Technique A = 302.47 ± 37.42 µm; Technique B = 180.45 ± 29.86 µm; Technique C = 147.34 ± 28.23 µm. The mean results for the Carestream 3600 scanner were: Technique A = 303.59 ± 40.20 µm; Technique B = 181.53 ± 29.61 µm; Technique C = 142.28 ± 35.33 µm. Technique C, used by both scanners, produced less volumetric discrepancies compared to the other techniques.

**Conclusions:**

The scanning technique had a statistically significant effect on the quality of the scan (*p*< 0.0001), whereas the scanner did not present any significant influence (*p*= 0.91).

## RESUMO

**Objetivo:**

o objetivo do presente estudo foi verificar e comparar a precisão de modelos digitais de uma arcada dentária completa obtidos utilizando-se dois tipos de *scanners* e três metodologias de digitalização.

**Métodos:**

um modelo de resina feito com impressão 3D foi digitalizado em um *scanner* de referência (Zfx Evolution - Zimmer Biomet, Palm Beach Gardens, FL) para se obter uma referência em 3D; o mesmo modelo de resina foi, então, digitalizado com dois *scanners* intrabucais diferentes (Zfx IntraScan e Carestream 3600 - CS 3600^®^, Carestream, Rochester, NY, EUA) utilizando: Técnica A (do dente #27 ao dente #17); Técnica B (do dente #11 ao dente #17 e, em seguida, do dente #21 ao dente #27); e Técnica C (do dente #22 ao dente #17 e, em seguida, do dente #12 ao dente #27 - o*software* MeshLab v. 1.3.3 foi, então, usado para mesclar as duas leituras). Em seguida, as imagens digitalizadas foram sobrepostas à imagem de referência, utilizando-se um *software,* e as discrepâncias volumétricas foram calculadas.

**Resultados:**

a média dos resultados para o *scanner* Zfx Intrascan foram: Técnica A = 302,47 ± 37,42 µm; Técnica B = 180,45 ± 29,86 µm; Técnica C = 147,34 ± 28,23 µm. A média dos resultados para o *scanner* Carestream 3600 foram: Técnica A= 303,59 ± 40,20 µm; Técnica B = 181,53 ± 29,61 µm; Técnica C = 142,28 ± 35,33 µm. A Técnica C, utilizada em ambos os *scanners*, produziu as menores discrepâncias volumétricas, quando comparada às outras técnicas.

**Conclusões:**

a técnica de digitalização teve um efeito estatisticamente significativo sobre a qualidade do modelo digital (*p*< 0,0001), enquanto o tipo de *scanner* usado não apresentou qualquer influência significativa (*p*= 0,91).

## INTRODUCTION

Successful orthodontic treatments rely largely on the careful treatment planning based on the accuracy of detail reproduction in dental impressions.^1^

Digital impressions are becoming increasingly popular because of many associated advantages, including faster turnaround time, real-time feedback for higher precision, a reduction in remakes, and improved workflow. Currently, three primary methods for producing 3D digital models are available: 1) laser-scans of plaster models of alginate impressions; 2) cone-beam computed tomography (CBCT) scans of alginate impressions or plaster models; 3) direct intraoral scans of dental arches.^2^ ^,^ ^3^

A fully digitized workflow offers several advantages: elimination of the traditional steps and the need for traditional impression materials, reducing the potential inaccuracies linked to contraction, expansion or deformation,^4-8^ and the deformities linked to deep bites or undercuts in the orthodontic brackets;^9^ in addition, facilitates the transfer of digital data to the technician, which leads to cost reduction for the dentist, technician, and patient.^5^ It can also improve patient comfort (especially those with an accentuated gag reflex). Moreover, intraoral scanning can be interrupted if necessary, to let the patient rest.^5^ There is also a reduction in chair time as the impression procedure is faster and more effective.^4^ ^,^ ^8^ Finally, the use of intraoral scanners eliminates the risk of wax bite distortion because bite registration is taken with the patient’s teeth in centric occlusion.^9^ Digital study models can thus potentially improve the workflow of a dental practice, ensuring a high degree of standardization.^4^

Several studies have been conducted to evaluate the reliability and validity of impressions fabricated using digital methods. A study carried out in 2013 used linear measurements to compare the accuracy of intraoral scans and traditional models:^6^ overestimations of the overall Bolton ratio (0.209 mm) and the anterior Bolton ratio (0.427 mm) by the digital method were found. There was a mean difference of 0.024 mm in tooth width measurements for the digital model (CI 95% = 0.006 - 0.041 mm), with a maximum difference for the left maxillary first molar (+0.117 mm) and a minimum difference for the left mandibular first molar (-0.003 mm). Another study by van der Meer et al^11^ used linear measurements to compare the accuracy, reliability and reproducibility of digital impressions obtained using three intraoral scanners. Differences ranging from -0.04 mm to + 0.16 mm were found between traditional and digital models when the CBCT scanner was used, and differences ranging from -0.24 mm to +0.07 mm were found between traditional models and intraoral scanned models. While the authors of both studies concluded that digital models obtained from intraoral scanning were valid and reliable for dental measurements and diagnostic purposes, they nevertheless theorized that, although not clinically significant, an error of 0.1-0.2 mm can have an important impact on a dental implant positioned using a digital model.^10^ ^,^ ^11^

**Figure 1:**
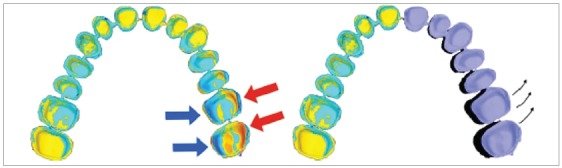
Deformations characterized by a systematic deviation, particularly evident in the most distal sector of the arch.

Other studies have evaluated the accuracy of various types of intraoral scanners, but the data registered by these studies were linear, and not volumetric, and refer to average measurements of relevant zones - they do not show the real volumetric discrepancy of a full arch. The authors pointed out that the 3D virtual model obtained from a digital impression showed deformations characterized by a systematic deviation particularly evident in the most distal segment of the dental arch, the last part to be scanned (Fig 1). 

These data seem to indicate that 3D digital models obtained from full-arch scans by intraoral scanners are sufficiently accurate for diagnostic evaluations and preliminary dental measurements;^1^ however, the sum of errors associated with the software matching processes makes the technique unsuitable for clinical applications such as lingual brackets indirect bonding, orthodontic appliances, surgical planning, or indirect splints.^7^ ^,^ ^10^

Thus, the aim of the current study was to verify and compare the accuracy of full-arch digital impressions fabricated using three scanning methodologies and two different intraoral scanners. The null hypothesis of this study was that the scanning technique has no effect on the accuracy of full-arch digital impressions.

## MATERIAL AND METHODS

A resin model of all the natural teeth of an upper dental arch (including elements from #17 to #27) was scanned in order to obtain a 3D reference impression. For this purpose, Zfx Evolution (Zimmer Biomet, Palm Beach Gardens, FL), a widely recognized as being a high precision reference scanner, was used.^12^ An independent laboratory specialized in measuring, designing and fabricating CAD/CAM structures to obtain a 3D digital reference (R data) was commissioned to acquire the reference model, using a fully automatic Zfx Evolution. In accordance with the VDI (Association of German Engineers) standards, the instrument quickly scans the entire arch with a margin of error inferior to 9 µm across a volume of 120 mm *x* 80 mm. Surfaces are recognized through a photometric technology: the LED light source of the scanner projects a total of 128 line pairs on the model’s surface. Acquisition is carried out by twin cameras with a resolution of 1296 x 964 pixels that scan the model placed on a rotating base on two axes, to guarantee that all details are registered. The data obtained can be saved on a standard STL file.

Then the model was scanned by two different intraoral scanners:

» Zfx IntraScan (Zimmer Biomet, Palm Beach Gardens): a lightweight handpiece scanner, connected to a notebook by means of a cable, useing confocal system to measure the distance between the scanner and the structure being scanned.
» Carestream 3600 (CS 3600^®^, Carestream, Rochester, NY, USA): a powerful structured LED light device.

The resin model was placed on a stable base on a worktable and scanned by an expert operator; three different techniques, commonly applied to register a patient’s bite, were used in order to acquire ten full-arch scans for a total of 60 acquisitions with a 5-minute lag between each scan. 

All the scans were carried out following the manufacturer’s instructions; they were executed on the same day and in the same place, in order to guarantee standardized and homogeneous conditions.

### Technique A (n = 10)

Scanning started at element #27 continuing along the entire arch up to element #17 (Data A).

### Technique B (n = 10)

Scanning started at element #11 continuing in the distal direction up to element #17; then, it started at element #21 and continued in the distal direction up to element #27 (Data B).

### Technique C (n = 10)

The scanner acquired the reference model in two steps. The first scan started at element #22 and continued in the distal direction until element #17. The second scan started at element #12 and continued up to element #27. The two scans were matched by the MeshLab software (v. 1.3.3), a powerful software for processing 3D scans (Data C).

### Measuring and comparing the 3D models

The STL files obtained were loaded into 3D metrology Geomagic software (Geomagic Control™, Geomagic, Morrisville, USA). The data acquired by each of the three techniques were superimposed on and aligned to the reference scan (Data R), using the best fit algorithm of the software.

The volumetric deviations (in the x, y, z axes) between each acquisition (A, B, C) and the reference data were calculated. The comparison software identified relevant discrepancies that assumed positive or negative values. The tridimensional differences were also visualized as a superimposed image with a color-coding, indicating expansion or contraction areas.

The majority of studies on full arch scans obtained by intraoral scanner consists of *in vitro* study aiming to identify the most accurate one.^13^ Therefore, an *in vitro* study was conducted in order to test different intraoral scanners and scanning techniques. 

Scanning was performed by an expert operator experienced in full arch methodology; the scanner requires 2-3 minutes to complete a full-arch acquisition.

### Statistical analysis

The mean of the absolute values of the discrepancies was obtained from the superimposition of each scan over the reference model. This value, expressed in µm, was then used for statistical analysis.

The mean discrepancy and the standard deviation of each data group were calculated to compare the three acquisition techniques. The mean values obtained represent the volumetric errors. Two-way factorial ANOVA was carried out to compare the differences between the data groups, using a significance level of α?#8197;= 0.05. The calculations were made using SPSS Statistics 22.0.0.0.

## RESULTS

When the scans were matched with the R-data, the mean results for the Zfx scanner were: Technique A = 302.47 ± 37.42 µm, Technique B = 180.45 ± 29.86 µm, Technique C = 147.34 ± 28,23 µm. The Carestream scanner results were: Technique A = 303.59 ± 40.20 µm, Technique B = 181.53 ± 29.61 µm, Technique C = 142.28 ± 35.33 µm.

Statistical analysis (two-way factorial ANOVA) was carried out to look for significant differences between the three techniques and the two different scanners.

Compared with Technique A, Technique B had an estimated difference of -122, a statistically significant level with a < 0.0001 *p*-value.

Compared with Technique A, Technique C had an estimated difference of -158, a statistically significant level with a < 0.0001 *p*-value.

The results showed that Technique A was the least accurate scanning technique, while Technique C was the most accurate one. The scanning technique influenced the quality of the scan (*p*< 0.0001), whereas the scanning system didn’t have any significant effect on it (*p*= 0.91). The interaction between the technique and the system used, which produced a *p*-value = 0.95, confirmed that the scanning system used did not influence the efficacy of the scan.

The three-dimensional discrepancies between the techniques and the reference scan were visualized as superimposed images, with a color code: colors ranging from yellow to red indicated positive (expansion) deviations, while colors ranging from blue to purple indicated negative (contraction) deviations. A visual analysis of the superimposed data revealed a net expansion in the most distal segment of the first quadrant (the last part of the dental arch that was scanned when Technique A was used). A more homogeneous pattern with alternating areas of contraction and expansion was associated with Technique B; fewer discrepancies were found in scans obtained using Technique C (Fig 3).

**Figure 2:**
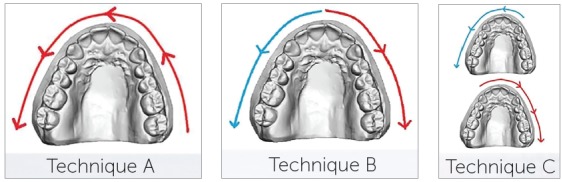
Three different scanning methodologies.

**Figure 3:**
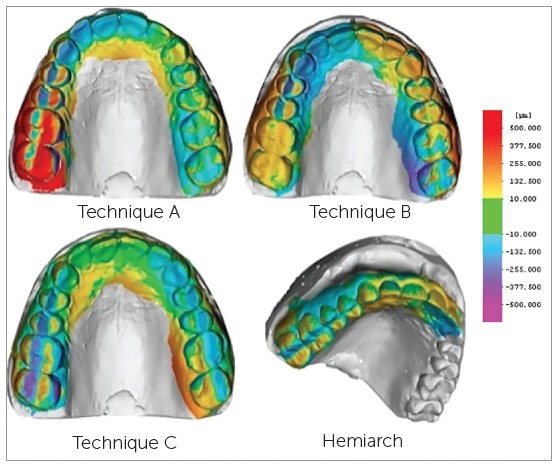
The three-dimensional discrepancies between the techniques and the reference scan were visualized as superimposed images, with a color code.

The mean discrepancies between the reference model and the right and left hemi-arches, obtained using Technique C, were also calculated: the values obtained for the right and left hemi-arches were 81.77 ± 30.0 and 80.71 ± 28.46, respectively.

## DISCUSSION

To our knowledge, this is one of the first studies aiming at comparing the accuracy of different scanning methodologies using different intraoral scanners. The ‘two-step’ scanning method, which matched a first scan from element #22 up to element#17 and a second scan from #12 up to element #27 (Technique C), was the most accurate technique, according to our analysis. It was followed by Technique B (scanning from element #11 up to element #17, and then from element #21 up to element #27); and then by Technique A (starting from element #27, continuing along the entire arch up to element #17). Results showed that there were statistically significant differences between the three techniques, and the null hypothesis was thus refuted.

Although the superimposition of the 3D models acquired using a virtual reference model and a dedicated software is considered a valid methodology and has been used in numerous studies on digital impressions,^5^ ^,^ ^14^ ^,^ ^15^ ^,^ ^16^ it does have some limits, as pointed out by Guth et al.^8^ Reference scanning inevitably produces errors, though minor, compared to real values. Further errors are inevitably introduced during the superimposition process by the best fit alignment algorithm of the Geomagic software and by the union of the scans of the hemi-arches (Technique C) using the MeshLab software. Aligning the scans makes it possible to attain positive and negative values between the reference data and the data being investigated. The best approximation of the discrepancy between the scans is represented by the absolute value of the positive and negative deviations.^8^

All these considerations have led us to the conclusion that, from a methodological point of view, this approach is the best way to compare scans and to determine which of the three methods is the most accurate. The results obtained cannot, however, be considered absolute values of full-arch scans.

An analysis of study results clearly showed that Technique A was the least accurate; this was evident even at the visual examination of the superimposed color code image. Considerable discrepancies compared to the reference scan were noted in the distal portion of the first quadrant, which was the last part of the arch to be scanned. The deformation was due to cumulative errors and a systematic deviation that worsened as the length of the arch being scanned lengthened. This can be explained by the coupling processes of the data by the software and by the sum of errors accumulated during the acquisition, which becomes more evident in longer scans. Other investigators^7^ ^,^ ^10^ ^,^ ^14^ ^,^ ^15^ ^,^ ^17^ reported similar results. Ender and Mehl^7^ have hypothesized that these errors will probably be avoided or reduced by future developments in intraoral scanner technology.

Some authors have hypothesized that smaller discrepancies, both in terms of the mean volumetric error and the visual assessment of the superimpositions by the software, can be obtained by using different scanning techniques since deformations, represented by contractions and expansions, would be smaller and more homogeneously distributed. By beginning the scanning process at the central incisors and continuing towards the most distal portions, initiating at the first quadrant and then moving towards the second quadrant (Technique B), the total length of the arch to be scanned is cut in half, and the matching software will produce less deformations. More accurate results could be attained by performing two separate scans (one from element #22 to element#17, and the other from element #12 to element #27), and then joining the two scans using a dedicated software. This hypothesis is in agreement with the finding that the cumulative error of matching data detected during acquisition is reduced by shortening the length of the arch to be scanned, even though the software used to combine the two scans (MeshLab) inevitably introduces errors.

There are very few studies on the accuracy of digital impression systems used for full-arch scans of natural teeth. In the first of two successive studies, Ender and Mehl reported errors of 49 ± 14.2 µm with respect to the reference scan when Cerec Bluecam system was used, and errors of 40.3 ± 14.1 µm when Lava C.O.S. scanner was used.^7^ ^,^ ^14^ ^,^ ^15^ Errors of 58.6 ± 15.8 µm were found when the Cerec system was used during the second study (carried out with a new reference scanner). Patzeltz et al^15^ reported mean accuracy values of 38 ± 14.3 µm (Lava C.O.S.), 49.6 ± 14 µm (iTero), 73.7 ± 26,6 µm (Zfx Intrascan) and 332.9 ± 64.8 µm (Cerec Bluecam).These values, which are smaller than those found in the current study, can be explained by the methodology used to measure the discrepancies in the reference scan. It is nevertheless important to remember that the mean values in the studies cited were expressed as linear measurements, whereas the mean discrepancy obtained by the current investigation is a volumetric value.

The findings outlined here confirm that measurements reported in this study should not be considered absolute values of the full-arch scans, but rather a mean to compare scans (obtained using different techniques or with different scanners).

The mean volumetric discrepancy obtained from the scan of a single hemi-arch (from element #22 to element #17 for the right hemi-arch; from element #12 to element #27 for the left one) was also calculated. The results obtained (81.77 ± 30.0 µm and 80.71 ± 28.46 µm respectively) are significantly lower compared to full-arch acquisitions. This result is consistent with findings indicating that limiting the part of the arch to be scanned to a single quadrant leads to better overlapping accuracy results, compared to conventional impressions, and to clinically acceptable values.^8^ ^,^ ^16^ ^,^ ^18^ ^-^ ^21^

We made use of a resin reference model and avoided using a plaster model because it tends to be sensitive to physical factors such as water and mechanical insults.

The scanning systems (Zfx Intrascan and Carestream 3600) used to acquire the scans of the hemi-arches are popularly used scanners that produced overlapping similar results.

Previous studies have used other measurement methods such as a coordinated measuring machine (CMM) and computed tomography machine, which are even more precise than a laboratory scanner in carrying out reference scans. This could be considered a limitation of this study, although the Zfx Evolution is widely considered a high precision reference scanner.^12^

The study was carried out *in vitro* in the attempt to produce standardized results of measurements taken in the same environment at the same time. Intraoral impressions taken from a real patient would probably be less accurate than the ones obtained in this study, because conditions such as the presence of saliva, different reflection indexes, and movement by the patient could affect the results. Moreover, these factors could slow down the scan and make the procedure more difficult.

Digital impressions acquired using intraoral instruments are becoming increasingly popular as the methods used to produce them are more accurate and user-friendly, with shorter learning curves and associated to patient comfort and satisfaction. Future studies should investigate the limits of intraoral scanners and scanning techniques. Up to the present moment, the combined use of both digital impression methods and conventional approaches continues to guarantee high quality results.

## CONCLUSIONS

» There were significant differences in accuracy between the three scanning techniques used to acquire full-arch digital impressions (*p*< 0.0001).
» The type of scanner used did not affect the accuracy of the scan (*p*= 0.91).
» The errors in the scans are proportional to the length of the hemi-arch scanned: errors were increased in longer hemi-arches, errors were reduced in shorter ones.
» Errors are still found when only a single hemi-arch is scanned.
